# Nitric oxide and histone deacetylases modulate cocaine-induced mu-opioid receptor levels in PC12 cells

**DOI:** 10.1186/2050-6511-13-11

**Published:** 2012-10-18

**Authors:** Warren Winick-Ng, Francesco Leri, Bettina E Kalisch

**Affiliations:** 1 Department of Biomedical Sciences, University of Guelph, Guelph, Ontario, N1G 2W1, Canada; 2 Department of Psychology, University of Guelph, Guelph, Ontario, N1G 2W1, Canada

**Keywords:** Cocaine, PC12 cells, Histone acetylation, Nitric oxide, Mu-opioid receptor

## Abstract

**Background:**

Cocaine exposure has been reported to alter central μ-opioid receptor (MOR) expression *in vivo*. The present study employed an *in vitro* cellular model to explore possible mechanisms that may be involved in this action of cocaine.

**Methods:**

To assess the effects of cocaine on MOR levels, two treatment regimens were tested in PC12 cells: single continuous or multiple intermittent. MOR protein levels were assessed by western blot analysis and quantitative PCR was used to determine relative MOR mRNA expression levels. To evaluate the role of nitric oxide (NO) and histone acetylation in cocaine-induced MOR expression, cells were pre-treated with the NO synthase inhibitor N^ω^-nitro-L-arginine methylester (L-NAME) or the non-selective histone acetyltransferase inhibitor curcumin.

**Results:**

Both cocaine treatment regimens significantly increased MOR protein levels and protein stability, but only multiple intermittent treatments increased MOR mRNA levels as well as *c-fos* mRNA levels and activator protein 1 binding activity. Both regimens increased NO production, and pre-treatment with L-NAME prevented cocaine-induced increases in MOR protein and mRNA levels. Single and multiple cocaine treatment regimens inhibited histone deacetylase activity, and pre-treatment with curcumin prevented cocaine-induced up-regulation of MOR protein expression.

**Conclusions:**

In the PC12 cell model, both NO and histone deacetylase activity regulate cocaine-induced MOR expression at both the transcriptional and post-transcriptional levels. Based on these novel findings, it is hypothesized that epigenetic mechanisms are implicated in cocaine’s action on MOR expression in neurons.

## Background

Endogenous opioid systems are involved in several aspects of cocaine addiction [1-5], and several studies have indicated that cocaine increases μ-opioid receptor (MOR) mRNA and peptide expression [6-11] in regions of the brain known to regulate incentive motivation and stress reactivity [12-14]. In rats, cocaine-induced increases in MOR mRNA expression have been consistently observed in the ventral striatum [15,16], a region of the brain critical to drug motivated behaviors [17,18]. Furthermore, PET studies in abstinent cocaine users have established correlations between elevations in MOR binding in mesocorticolimbic areas and intensity of cocaine cravings [19-21]. These data suggest the importance of elucidating the molecular mechanisms through which cocaine alters MOR levels in the central nervous system.

The experiments reported in this manuscript were designed to investigate two possible, and related, mechanisms. First, cocaine administration elevates concentrations of nitric oxide (NO) in the rat brain [22,23], and both cocaine and NO increase levels, and binding activity, of members of the activator protein 1 (AP-1) transcription factor family [24-32]. Because the promoter region of the MOR gene contains consensus sequences for AP-1 transcription factors [33], it is possible that cocaine modulates MOR expression via alterations in NO and AP-1 activity. Second, NO also decreases the activity of histone deacetylases (HDACs) [34,35], enzymes implicated in the behavioral effects of cocaine in rats [36,37], as well as in morphine-induced MOR expression [38,39]. HDACs affect chromatin structure through the removal of acetyl groups from histones [40-42], and thus contribute to gene transcription [43-47]. Therefore, it is also likely that cocaine modulates MOR expression via alterations in histone acetylation.

These experiments employed PC12 cells to investigate the role of NO and HDACs in cocaine-induced alterations of MOR expression. This *in vitro* cellular model was selected because PC12 cells express the MOR gene [48-50], their NO pathway has been fairly well characterized [51-54], and they are sensitive to changes in HDACs activity [55]. Three main results were obtained. First, cocaine increased MOR protein expression and protein stability after both single continuous and multiple intermittent treatment regimens, but only the multiple intermittent treatment regimen increased expression of MOR and c-fos mRNAs, as well as AP-1 binding activity. Second, NO was identified as an important modulator, as cocaine increased NO production, and the NO synthase (NOS) inhibitor N^ω^-nitro-L-arginine methylester (L-NAME) attenuated cocaine-induced increases in MOR protein and mRNA expression. Third, it was found that cocaine decreased HDACs activity, and inhibition of histone acetyltransferase (HAT) attenuated cocaine-induced increases in MOR protein expression following both treatment regimens.

## Methods

### Materials

Dulbecco's modified Eagle medium (DMEM), horse serum, gentamycin, DNAse I, Oligo dT, Superscript II, primers, Platinum Taq and Lipofectamine 2000 were purchased from Invitrogen (Mississauga, ON, Canada) and fetal bovine serum (FBS) was obtained from HyClone Laboratories (Logan, UT, USA). Cocaine HCl was purchased from Dumex (Toronto, ON, Canada), L-NAME, curcumin, and mouse monoclonal anti-α-tubulin were purchased from Sigma Aldrich (St. Louis, MO, USA). The complete mini tablets were purchased from Roche Diagnostics (Laval, QC, Canada), the sodium dodecyl sulfate (SDS) sample buffer, DTT, and protein standards were obtained from New England Biolabs (Ipswich, MA) and the polyclonal MOR antibody was from Abcam (Cambridge, MA, USA) or Santa Cruz Biotechnology Inc. (Santa Cruz, CA, USA). Luminol was also purchased from Santa Cruz. Hybond-C blotting membranes, sheep anti-mouse IgG and enhanced chemiluminescence (ECL) kit were obtained from Amersham/GE Health Care (Piscataway, NJ, USA), poly-D-lysine was from BD Biosciences (Mississauga, ON, Canada) and 4,5-diaminofluorescein diacetate (DAF-2 DA) was purchased from Calbiochem (San Diego, CA, USA). Syber Green PCR master mix was obtained from Qiagen (Toronto, ON, Canada) and the HDAC Assay kit was from Active Motif (Carlsbad, CA, USA). The PathDetect pAP-luciferase reporter plasmid was obtained from Stratagene (La Jolla CA, USA) and the Luciferase Assay and Galacto-Light (Tropix) kits were from Promega (Madison, WI, USA) and Applied Biosystems (Bedford, MA, USA), respectively. All other chemicals were molecular or electrophoresis grade and obtained from Fisher Scientific (Ottawa, ON, Canada) or DiaMed Laboratories (Mississauga, ON, Canada).

### Cell culture, viability and treatments

PC12 cells were maintained in DMEM containing 5% FBS, 5% horse serum and 50 μg/mL gentamycin at 37^o^C in 5% CO_2_. To evaluate the effects of cocaine, NO synthase (NOS) inhibitors, and curcumin on MOR protein and mRNA levels, cells were plated on Corning® 60 mm dishes at a density of 1.0 million cells per plate for protein, and 1.5 million cells per plate for RNA. For the AP-1 study, PC12 cells were plated on 12-well culture dishes at a concentration of 2.0 x 10^5^ cells per well. For NO production imaging, PC12 cells were plated on 6-well culture dishes containing poly-D-lysine coated coverslips at a concentration of 2.0 x 10^5^ cells per well. For nuclear extraction, PC12 cells were plated on 100 mm culture dishes at a concentration of 4.0 x 10^6^ cells per plate. All plating was performed 24h prior to any treatment.

The effects of cocaine were determined by exposing PC12 cells to various concentrations of cocaine using two different treatments. The doses of cocaine selected for this study (10, 100, and 500 μM) were based on previous reports investigating the effects of cocaine on morphological changes and proto-oncogene expression in PC12 cells [56]. Two treatment regimens were chosen based on previous findings indicating that different exposure patterns can differentially affect MOR binding affinity and receptor density in several regions of the rat brain [57,58]. These treatments were: single continuous treatment (SCT) or repeated intermittent treatment (RIT) (see Table 1). The latter regimen included 3 daily treatments, each lasting 30 min, separated by 60 min exposures to cocaine-free media. Cells were harvested 72 h after the beginning of treatment, except where otherwise indicated.

PC12 cell viability was assessed in control cells and those exposed to 500 μM *SCT* or 100 μM *RIT* with cocaine for 72 h by the reduction of 3-(4,5-dimethylthiazole-2-yl)-2,5-diphenyltetrazolium bromide (MTT) as described by Cheung et al. [59]. Culture media was replaced with media containing MTT (0.5mg/mL final concentration) and the cells were incubated at 37°C for 30 min. The reduced formazan product was lysed from the cells using a 100% dimethylsulfoxide solution and the absorbance was subsequently measured at 570nm using the FLUOstar Optima plate-reader (BMG, Fisher Scientific).

The effect of NOS and HAT inhibitors was determined by pre-treating PC12 cells with inhibitor, for 1 h prior to cocaine exposure. We determined previously that NGF increases NOS activity and NO production in PC12 cells and that this increase is attenuated when cells are pretreated with 20 mM L-NAME [53]. Therefore, cells were pretreated with this dose of L-NAME to examine the involvement of NO in the cocaine-induced expression of MOR. Cells were also pretreated with 1, 3 or 5 μM of the non-selective HAT inhibitor curcumin, based on a previous report by Siddiqui et al. [60], which explored the impact of curcumin on oxidative stress in PC12 cells. Curcumin was selected for these experiments because it inhibits HAT in both *in vitro* and *in vivo* models [61-63] and it modulates cocaine place preference in rats [36]. For *RIT*, each 30 min cocaine treatment was followed by the addition of PC12 cell culture media containing only L-NAME (20 mM) or curcumin (1, 3, 5 μM).

### Immunoblot analysis

Control and treated PC12 cells were lysed in 250 μL of radioimmunoprecipitation assay (RIPA) buffer (final concentration: 50 mM Tris, 150 mM NaCl, 1% NP-40, 0.25% sodium deoxycholate, 0.5% SDS, 1 mM each of EDTA, sodium fluoride, sodium orthovanadate and protease inhibitor (1 Complete Mini Tablet (Roche Diagnostics)/10 mL], pH 7.4). Samples were rocked on ice for 15 min, sonicated, centrifuged at 17 530 g for 15 min and the protein content of the supernatant determined by the method of Bradford [64]. Cell lysates (100 μg) were then boiled in SDS sample buffer (final concentration: 62.5 mM Tris-HCl; pH 6.8, 2% SDS, 42 μM DTT, 10% glycerol and 0.01% phenol red) and loaded onto a 10% SDS/polyacrylamide gel.

Following electrophoresis, proteins were transferred onto nitrocellulose membranes (Hybond-C) using a Trans-blot semidry transfer unit (Bio-Rad Laboratories, Mississauga, ON, Canada) with transfer buffer (final concentration: 28 mM Tris, 39 mM glycine and 20% methanol, pH 9.2). Membranes were blocked in 2.5% or 5% non-fat milk in tris-buffered saline (TBS) containing 0.1% Tween-20 (TBS-T) for 1 h. Blots were then incubated in 1:750 rabbit MOR antibody in 1% bovine serum albumin (BSA) in TBS-T for 2 h (Abcam), or in 1:200 rabbit MOR antibody in 1% non-fat milk in TBS-T (Santa Cruz) overnight. Antibody detection was achieved using 1:2500 horseradish peroxidase-conjugated donkey anti-rabbit IgG in either 1 % BSA or 5% non-fat milk in TBS-T for 1 h, followed by ECL or luminol.

Membranes were scanned using the STORM 860 (Molecular Dynamics, subsidiary of Amersham) for ECL, or the Fluorchem 9900 imaging system (Alpha Innotech, Santa Clara, CA, USA) for luminol. Bands were analyzed densitometrically using Imagequant (Molecular Dynamics) or Fluorchem 9900 software. Blots were stripped with 62.5 mM Tris, pH 6.7, containing 2% SDS and 100 mM 2-mercaptoethanol at 50^o^C for 20 min. Membranes were then rinsed in TBS for at least 4 h before blocking with 5 % milk in TBS-T for 1 h and reprobing with 1:50 000 mouse monoclonal anti-α tubulin antibody overnight. Blots were then exposed to 1:2500 goat anti-mouse IgG-horse radish peroxidase conjugated secondary antibody, in 5% milk in TBS-T and the protein bands visualized as described above.

### MOR half-life analysis

Following 72 h of treatment, control and *SCT* or *RIT* PC12 cells were exposed to 10 μg/mL cycloheximide [65], a *de novo* protein translation inhibitor, for 4, 8, 12, 24 and 48 h. Cells were then lysed in 250 μL RIPA buffer, and western blot analysis for MOR and α-tubulin was performed as described above.

### Analysis of NO production

NO production was assessed using the fluorescent probe DAF-2 DA. PC12 cells were treated with cocaine (100 or 500 μM) in the presence or absence of 20 mM l-NAME for 3 days, washed once with media and loaded with 10 μM DAF-2 DA in 1 mL culture media. Following 2 h of incubation cells were washed 4 times with 2 mL media and DAF-2 fluorescence was visualized using an Olympus IX-81 fluorescence microscope (excitation at 488 nm, emission at 520 nm) with an Olympus LucPlan FL 0.40 aperture lens (at 20 x magnification) in phosphate buffered saline at room temperature. Digital images were captured using a Cascade 512F camera (Photometrics, Tucson, AZ, USA), and processed in Q-Capture and Adobe Photoshop 5.0.

### qPCR and PCR analysis

In our previous studies of gene expression in PC12 cells, changes in mRNA typically occurred 24 to 36 h prior to changes in protein expression [53]. Therefore we initially examined MOR mRNA following 48h of *SCT* and *RIT* with cocaine. Control and treated PC12 cells were lysed in 1 mL of Trizol reagent to obtain total RNA. The extracted RNA was treated with DNase I and reverse transcribed using superscript II with oligo-dT as the primer for 75 min at 43°C [53]. The resulting cDNA was then used for real time PCR (qPCR) using a LightCycler (Roche). qPCR was performed using 1 μL cDNA and 9 μL of QuantiTect SYBR Green PCR master mix. There was a 15 min incubation period at 95°C prior to the first cycle, and a melting curve was obtained for each sample following the final cycle [65]. Primer pairs and cycling conditions were: β-2-microglobulin (Genbank Accession number NM_012512): 5’ primer: 5’-TGACCGTGATCTTTCTGGTG-3’ and 3’ primer: 5’-ATCTGAGGTGGGTGGAACTG-3’, 45 cycles of 95°C: 15 s, 55°C: 25 s, 72°C: 15 s; MOR coding region (Genbank Accession Number U02083.1): 5’ primer: 5’-CTGTGTGTTACGGCCTGATG-3’ and 3’ primer: 5’-ATGCAGAAGTGCCAGGAAAC-3’, 55 cycles of 95°C: 15 s, 52°C: 25 s, 72°C: 15 s. After each cycle, fluorescent activity was determined, and a final crossing point (threshold cycle, C_T_) was calculated. Steady-state MOR mRNA levels relative to β-2 microglobulin were determined with RelQuant software

To qualitatively observe the effect of cocaine on *c-fos* levels, total RNA was extracted from control and treated PC12 cells as described above 0.5, 1 or 2 h after the final 30 min 100 μM cocaine treatment on the first day of *RIT*. Following reverse transcription, 5 μL cDNA was combined with 45 μL of master mix containing (final concentration): 15 mM MgCl_2_, 10 mM dNTPs, 5 μM of forward and reverse primers, 10 x PCR buffer, 0.2 μL of Platinum Taq and water [53,65]. Each PCR was performed with an initial 2 min, 95°C strand separation and a final 2 min, 72°C elongation. Primer pairs and cycling conditions were: β-actin (Genbank Accession number NM_031144): 5’ primer 5’-TCATGAAGTGTGACGGTTGACATCCGT-3’ and 3’ primer 5’-CCTAGAAGATTTGCGGTGCACGATG-3’, 30 cycles of 95°C: 30 s, 55°C: 30 s, 72°C: 45 s; *c-fos* coding region (Genbank Accession number NM_022197 XM_234422): 5’ primer: 5’-ACGCGGACTACGAGGCGTCA-3’ and 3’ primer: 5’-GCTCTGGTCTGCGATGGGGC-3’, 40 cycles of 95°C: 30 s**,** 55°C: 30 s**,** 72°C: 45 s. PCR products were separated on a 1.5% agarose gel stained with ethidium bromide. Fragments were visualized using the Pharmacia Biotech ImageMaster VDS, and images captured were processed in Microsoft Office Picture Manager. To quantify steady-state mRNA levels qPCR was performed as described above and analysis of *c-fos* levels relative to β-2 microglobulin determined using RelQuant software.

### AP-1 luciferase activity

PC12 cells were fed with antibiotic free media 30 min prior to transfection. For each well 1.0 μg luciferase reporter plasmid containing 7 AP-1 transcription factor binding elements was incubated with 0.5 μg of a pSV- β-galactosidase (β-gal) plasmid and 2 μL Lipofectamine2000 in 100 μL OptiMEM for 30 min at room temperature [51]. The mixture was added to PC12 cells and the plates returned to the cell culture incubator for 5 h. The transfection medium was replaced with regular PC12 culture medium and the cells returned to the incubator overnight. Following 6 h of *SCT* or *RIT* with cocaine or 50 ng/mL NGF (used as a positive control for AP-1 activation [51]) treatment, PC12 cells were lysed with 200 μL 1X passive lysis buffer (Promega). Lysates were incubated for 30 min at 4°C, followed by centrifugation at 12 000 x *g* for 2 min. Duplicate 20 μL samples of the supernatant were then transferred to a 96 well plate. To measure luciferase activity, 50μL of Luciferase Assay Reagent was added to each well, and after a 2 s delay, luciferase activity was read for 10 s. Luciferase activity was an indicator for AP-1 plasmid activation. Duplicate samples from the same lysate were used to measure β-gal activity with the Galacto-Light kit. First, 25 μL of galacton (1:100 with reaction buffer diluent) was added to each well and the samples were incubated for 30 to 60 min. This was then followed by the addition of 50μL of accelerator (provided in the Galacto-Light kit) to each well, and after a 2 s delay, β-gal activity was read for 1 s. Luciferase and β-gal activity was measured using the FLUOstar Optima plate-reader luminometer (BMG, Fisher Scientific).

### Nuclear extraction and HDAC enzyme activity assay

Since *c-fos* expression and AP-1 activity were altered within 24 h of treatment, it was hypothesized that cocaine would alter HDAC activity within a similar time frame. Following 24 and 36h of *SCT* and *RIT* with cocaine, PC12 cells were harvested in nuclear extraction buffer A (final concentration: 10 mM HEPES; pH 7.9, 1.5 mM MgCl_2_, 10 mM KCl, 0.5 μM DTT and protease inhibitor (1 complete MINI tablet/10 mL)) containing phosphatase inhibitors (1mM each of sodium fluoride, sodium orthovanadate). Cells were lysed using a 27.5 gauge needle, and the nuclei were pelleted by centrifugation at 14 000 x *g* for 5 min at 4°C. The supernatant was discarded and the pellet was rinsed in buffer A. Cells were again centrifuged at 14 000 x *g* for 5 min at 4°C, the supernatant discarded and the pellet re-suspended in nuclear extraction buffer B (final concentration: 20 mM HEPES; pH 7.9, 1.5 mM MgCl_2_, 420 mM KCl, 0.5 μM DTT, 25% glycerol, 2 mM EDTA, 1mM each of sodium fluoride, sodium orthovanadate, and protease inhibitor (1 complete MINI tablet/10 mL)) and kept on ice for 15 min. Following the addition of nuclear extraction buffer C (final concentration: 20 mM HEPES; pH 7.9, 0.5 μM DTT, 25% glycerol, 0.2 mM EDTA, 1mM each of sodium fluoride, sodium orthovanadate, and protease inhibitor (1 complete MINI tablet/10 mL)), samples were centrifuged at 10 000 x *g* for 5 min. The nuclear fraction in the supernatant was quantified using the method of Bradford [64].

Samples were used to detect HDAC enzyme activity using an HDAC assay kit according to the manufacturer’s recommendations. Briefly, nuclear extracts (7 μg) were added to a 96-well half-volume black plate, mixed with HDAC assay buffer and HDAC substrate (final concentration: 100 μM), and incubated at 37°C for 50 min. Following incubation, HDAC reactions were halted using HDAC developer (containing 2 μM Trichostatin A, final concentration: 1 μM) and incubated at room temperature for 12 to15 min. Fluorescence was detected using the FLUOstar Optima plate reader with an excitation wavelength at 350 nm and an emission wavelength at 460 nm.

### Data Analysis & Statistics

For western blot analysis, each band was analyzed densitometrically as described previously [52,65]. To account for variability between blots, the densitometric value for each individual MOR band was expressed as a fraction of the total amount of MOR protein present on the entire blot (ie. sample MOR density/sum of density of all MOR bands on blot). The same analysis was carried out for α-tubulin and then MOR protein values were normalized to α-tubulin values from the same sample.

For qPCR analysis, relative mRNA levels were determined using the delta-deltaCt method of analysis. A threshold cycle (C_T_) was determined for each data point. A ratio was then determined for each sample using by the following formula:

(1) $$\text{Ratio}=2^{-\left[\text{CT}\left(\text{GENE}\right)-\text{CT}\left(\beta 2\text{M}\right)\right]}$$

where C_T(GENE)_ was the gene of interest, and C_T(β2M)_ was the C_T_ for β-2-microglobulin. Each data point was then expressed relative to the control sample.

In order to determine HDAC enzyme activity, a standard curve was prepared using known HDAC assay standard dilutions. The amount of fluorescence recorded was then extrapolated to the pM amount of product formed (PF) from the standard curve. The specific activity (SpA) in pM/min/mg was then determined using the PF, incubation time in min (IT), and mass of nuclear extract in mg (mnx), by using the following formula:

(2) $$\text{SpA}=\left(\text{PF}/\text{IT}\right)/\text{mnx}$$

To determine relative luciferase activity, each sample was expressed as a percentage of the total amount of luciferase activity detected for each sample set. Luciferase activity was then normalized to β-gal activity within the same sample.

Data are representative of 5 independent experiments (except where otherwise indicated) and are presented as the mean ± standard error of the mean (SEM). Data were assessed for normality and homogeneity of variance and statistical analysis was carried out using a one or two-way analysis of variance (ANOVA). In the case of a one-way ANOVA, analysis was followed by Dunnett’s test or the Tukey-Kramer Multiple Comparisons test to determine which groups were significantly different. One-way ANOVA analysis was carried out for protein and RNA analysis of MOR levels following cocaine treatment, or for experiments with L-NAME pretreatment. For RNA analysis with cocaine and L-NAME, the data did not pass the Bartlett test for homogeneity of variance, therefore a Kruskal-Wallis non-parametric ANOVA was performed. For two-way ANOVA with interaction, analysis was followed by Bonferroni’s multiple comparisons t-test to determine which groups were significantly different. This analysis was used to determine significant changes in MOR protein expression following cocaine and curcumin treatments, and used to determine significant changes in *c-fos* mRNA expression following cocaine treatments over time. For two-way ANOVA without interaction, analysis was followed by contrast analysis to determine which groups were significantly different, and was used to determine significant changes in histone deacetylase activity following cocaine administration. Mean values were considered different if p<0.05.

For the protein half-life and AP-1 experiments, following western blot and luciferase analysis as described above, data were assessed for normality and homogeneity of variance. Due to a large disparity between variances, the natural log was taken for each data point. For the AP-1 luciferase analysis experiment, statistical analysis was carried out using a one-way ANOVA. Following transformation in the protein half-life experiment, data were analyzed using an analysis of co-variance (ANCOVA) to determine the effect of each treatment, as well as significant differences that may exist between the slopes of the resulting regression lines. The original raw data was also used to perform an ANCOVA, and, where possible, the resulting regression line was used to determine an approximate MOR half-life for each treatment. Mean values were considered different if p<0.05.

Data analysis was carried out using GraphPad InStat for one way ANOVA analysis, GraphPad Prism 4.0 or SAS 9.2 (SAS Institute, Cary, NC) for two-way ANOVA analysis with interaction, and SAS 9.2 for two-way ANOVA analysis without interaction, as well as ANCOVA analysis.

## Results

### Cocaine modulates MOR protein and mRNA levels, and protein half-life

**Figure 1:**
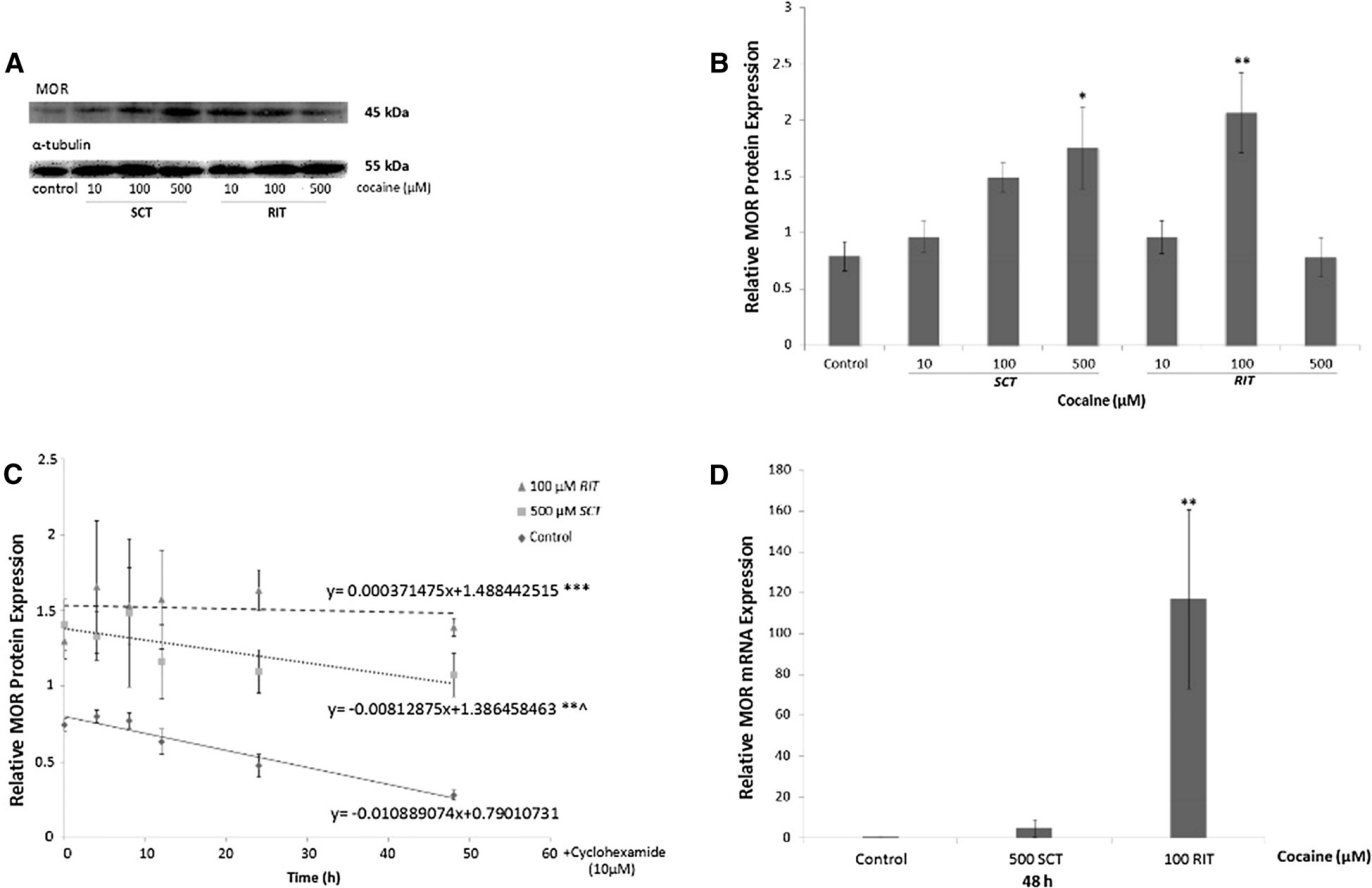
***Effect of cocaine on MOR protein and mRNA expression in PC12 cells.*** (**A**) Representative immunoblots obtained from lysates of control (untreated) and continuous (*SCT*) or intermittent (*RIT*) 10, 100, 500 μM cocaine treated PC12 cells separated by SDS-PAGE and transferred to nitrocellulose membranes. The top panel represents total MOR expression and the bottom panel shows α-tubulin levels from the same blot. (**B**) Densitometric analysis of MOR protein levels relative to α-tubulin levels in cocaine-treated cells revealed a significant increase in MOR expression following 500 μM *SCT* or 100 μM *RIT* compared to control. (**C**) Cell lysates were obtained from cells exposed to 500 μM *SCT* or 100 μM *RIT* with cocaine for 72 h, followed by 10 μg/mL cycloheximide for 4, 8, 12, 24 and 48 h. Densitometric analysis of MOR protein expression normalized to α-tubulin revealed an approximate half-life of 36.3 h in control (untreated) cells, and a significant change in the slope of protein decay following both cocaine treatment regimens. Protein half-life could not be estimated for *RIT*, but an increase in the predicted half-life to 85.3 h was calculated for *SCT*. (**D**) Quantitative PCR (qPCR) analysis of MOR mRNA levels relative to β-2 microglobulin in control and cocaine treated cells. Relative to control, 100 μM of *RIT* with cocaine significantly increased MOR mRNA levels while 500 μM of *SCT* had no effect. Results are representative of at least 5 independent experiments and the data are presented as mean ± SEM (*p<0.05, **p<0.01, ***p< 0.001, **^p=0.001).

The effect of cocaine on MOR protein levels was evaluated in extracts of control and treated (see Table 1 for treatment details) PC12 cells using western blot analysis. Figure 1A displays a representative immunoblot of MOR levels (upper panel) and corresponding α-tubulin levels (lower panel) obtained from cells treated with *SCT* or *RIT* with 10, 100 or 500 μM cocaine for 72 h. Densitometric analysis (Figure 1B) revealed a significant increase in MOR protein levels [F(6,33)= 5.75] relative to control in cells exposed to 500 μM *SCT* (p<0.05) or 100 μM *RIT* (p<0.01) cocaine. These treatments did not compromise PC12 cell viability: metabolic activity assessed with the MTT reduction assay indicated that, relative to control, there was only a modest decrease in activity in cocaine-treated cells (100 μM RIT: 90.1 ± 4.2 % of control, n=4; 500 μM SCT: 93.4 ± 2.9 % of control, n=4), which was not statistically significant. On the basis of these data, 500 μM *SCT* and 100 μM *RIT* cocaine were used in subsequent experiments.

**Table 1:** *Cocaine treatment regimens*

**Regimen**	**Description**
*SCT*	A single dose of cocaine added to the cell culture media and not removed over the entire time-course
*RIT*	Three daily intermittent 30 min treatments applied to the cells separated by 1 h of regular cell culture media.

PC12 cells were either given a single continuous treatment (SCT) or a repeated intermittent treatment (RIT) of cocaine.

To determine whether the cocaine-induced increase in MOR protein level was due to an increase in protein stability, protein extracts were obtained from cells treated for 72 h with 500 μM *SCT* or 100 μM *RIT* cocaine followed by 10 μg/mL cycloheximide for 4, 8, 12, 24 or 48 h. Densitometric analysis of MOR protein levels relative to α-tubulin revealed an estimated MOR half-life of 36.3 h in control (untreated) cells (Figure 1C), and a statistically significant effect of cocaine [F(2, 103)= 13.94, p<0.0001] and time (h) [F(1, 103) = 17.11, p<0.0001]. Both treatment regimens significantly changed the slope of protein decay compared to control, indicating that cocaine increased MOR half-life. Although it was not possible to calculate a predicted half-life for the cells treated with 100 μM *RIT* cocaine, MOR protein half-life following exposure to 500 μM *SCT* was estimated to be 85.3 h.

The effect of cocaine on MOR mRNA levels was also examined (Figure 1D). RNA was isolated from cells treated for 48 h with either 500 μM *SCT* or 100 μM *RIT* cocaine and levels of MOR mRNA relative to β2-microglobulin were assessed by reverse transcriptase (RT)-real time PCR (qPCR) analysis. Compared to control, a statistically significant increase in relative MOR mRNA levels was detected in cocaine-treated cells following *RIT* [F(2,15)= 6.80, p<0.01]*,* but not *SCT*.

### Effect of cocaine on nitric oxide, and its role in MOR protein and mRNA expression

**Figure 2:**
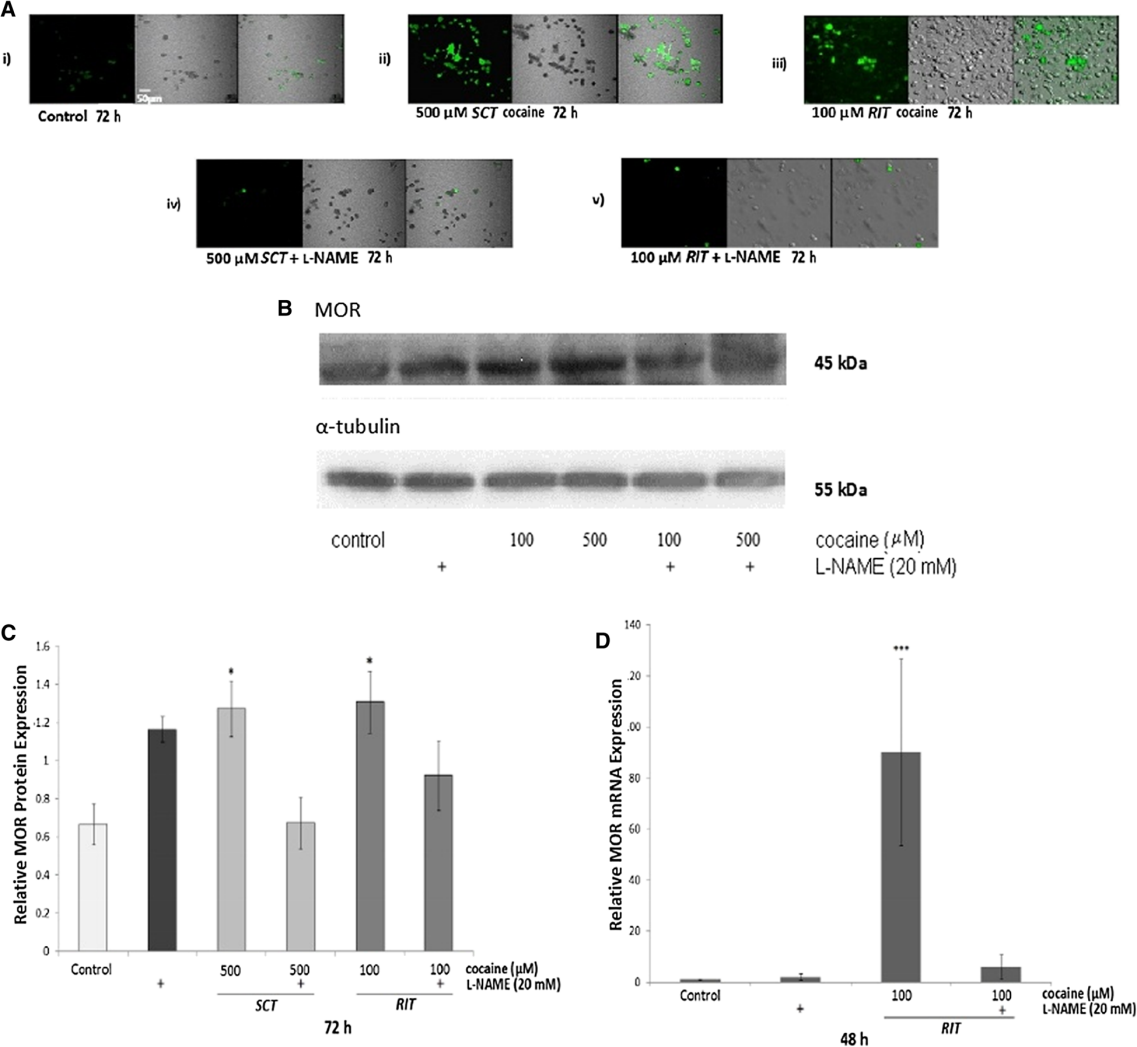
***Effect of cocaine on NO levels, and NOS inhibition on MOR protein and mRNA levels.*** (**A**) Representative images depicting DAF-2 fluorescence in (i) control (untreated) cells and cells treated with (ii) 500 μM *SCT*, or (iii) 100 μM *RIT* with cocaine for 72 h. The left column depicts fluorescence, the middle column is the corresponding Nomarski differential interference contrast image, and the right column is the overlay of these two images. Basal fluorescence was detected in control treated PC12 cells. Following exposure of cells to 500 μM *SCT* and 100 μM *RIT*, fluorescence intensity increased. The cocaine-induced increase in DAF-2 fluorescence was prevented when cells were pretreated with 20 mM L-NAME in both treatment regimens (iv and v). (**B**) Representative immunoblot of MOR protein (upper panel) and α-tubulin (lower panel) levels obtained from lysates of control (untreated) and cocaine-treated PC12 cells grown in the presence or absence of 20 mM L-NAME. Cells were exposed to 500 μM *SCT* or 100 μM *RIT* with cocaine for 72 h. (**C**) Densitometric analysis of MOR protein expression relative to α-tubulin in control, cocaine and L-NAME treated cells. Relative to control, cocaine (*SCT* and *RIT*) significantly increased relative MOR protein levels and this increase was prevented in cells pretreated with L-NAME. (**D**) qPCR analysis of MOR mRNA expression relative to β-2 microglobulin in control, cocaine (100 μM *RIT*) and L-NAME (alone or in combination with 100 μM *RIT* cocaine) treated PC12 cells. Relative to control, 100 μM *RIT* of cocaine significantly increased MOR mRNA, and this increase was prevented by pre-treatement with 20 mM L-NAME. Results are representative of at least 5 independent experiments and the data are shown as mean ± SEM (*p<0.05, ***p<0.001).

To examine the effect of cocaine on NO production, PC12 cells were treated with 500 μM *SCT* or 100 μM *RIT* for 72 h and diaminofluorescein-2 (DAF-2) fluorescence was examined. A low level of DAF-2 fluorescence, indicative of NO production, was observed in untreated PC12 cells (Figure 2A). DAF-2 fluorescence was increased in cocaine-treated cells with both treatments, and this increase was prevented when cells were pretreated with the non-selective NOS inhibitor L-NAME (20 mM).

In the subsequent experiment, PC12 cells were treated with 20 mM of L-NAME prior to 500 μM *SCT* or 100 μM *RIT* cocaine. Representative western blots depicting MOR and α-tubulin levels in extracts obtained from cells treated with cocaine in the presence or absence of L-NAME are presented in Figure 2B, and densitometric analysis of MOR expression relative to α-tubulin is depicted in Figure 2C. Relative to control, a significant increase in MOR protein levels was observed in cells treated for 72 h with either 500 μM *SCT* or 100 μM *RIT* cocaine [F(5,26)= 3.43, p<0.05 for both treatments]. This increase was not observed in cells pretreated with L-NAME.

The final experiment assessed the effect of 20 mM L-NAME pretreatment on MOR mRNA levels increased following 100 μM *RIT* cocaine. Compared to control, qPCR analysis (Figure 2D) revealed that cocaine significantly increased relative MOR mRNA levels (Kruskal-Wallis statistic= 15.36, p<0.001), and that this was prevented by pretreatment with L-NAME.

### Effect of cocaine on c-fos mRNA levels and AP-1 binding

**Figure 3:**
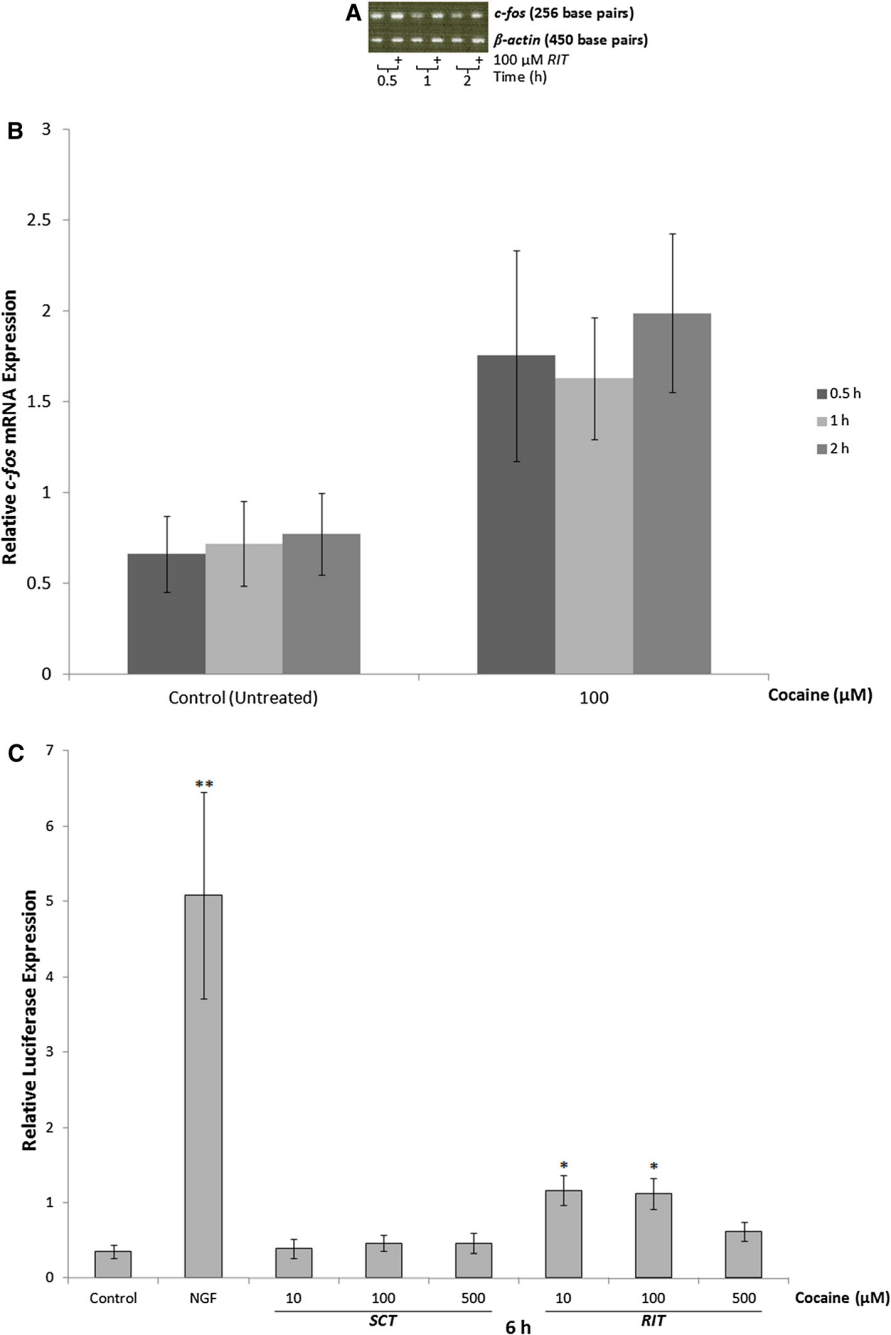
***Effect of cocaine on c-fos mRNA levels and AP-1 activity.*** (**A**) Representative ethidium bromide-stained gel showing amplification of *c-fos* (upper band) and β-actin (lower band) transcripts obtained from RNA extracted from control and cocaine-treated (100 μM *RIT*) PC12 cells, and harvested 0.5, 1, or 2 h after the last of three intermittent cocaine treatments. Qualitative assessment indicated that compared to control, cocaine exposure increased levels of *c-fos* mRNA at all of the time-points examined. (**B**) Quantitative PCR (qPCR) analysis of *c-fos* mRNA levels relative to β-2 microglobulin in control and cocaine treated cells. Relative to control, 100 μM of *RIT* with cocaine significantly increased *c-fos* mRNA levels [F(1,21)=11.41, p<0.01]. (**C**) PC12 cells were transiently transfected with a luciferase-reporter plasmid containing 7 AP-1 transcription factor binding sites prior to treatment with 50 ng/mL NGF or cocaine (10, 100, or 500 μM *SCT* or *RIT*) for 6 h. Luciferase activity in each sample was normalized to its corresponding β-gal activity. Relative to control, there was a significant increase in luciferase activity in PC12 cells treated with NGF, or 10 or 100 μM *RIT* with cocaine. Results are representative of at least 5 independent experiments and data are presented as the mean ± SEM (*p<0.05, **p<0.01).

Qualitative assessment of *c-fos* levels in PC12 cells treated with 100 μM *RIT* cocaine for 0.5, 1 or 2 h, revealed increases in steady-state *c-fos* mRNA (Figure 3A) relative to control. This was confirmed by qPCR analysis (Figure 3B) which revealed cocaine significantly increased relative *c-fos* mRNA levels [F(1,21)=11.41, p<0.01]. No increase in *c-fos* was detected following *SCT* cocaine. To investigate the effect of cocaine on AP-1 activity, PC12 cells were transiently transfected with a plasmid containing 7 AP-1 binding elements upstream from a luciferase reporter gene. The day after transfection, cells were exposed to 10, 100 or 500 μM *SCT* or *RIT* cocaine and harvested 6 h after the start of treatment (Figure 3C). As a positive control, other cells were treated with 50 ng/mL nerve growth factor (NGF) for 6 h [51]. Although modest compared to the NGF-mediated increase in AP-1 activity, both 10 and 100 μM *RIT* cocaine significantly increased AP-1 activity compared to control [F(7,33)= 10.181, p<0.01 for NGF, p<0.05 for cocaine]. No increase in luciferase activity was detected following *SCT* at all of the doses tested.

### Effect of cocaine on HDACs, and the role of histone acetylation in MOR protein expression

**Figure 4:**
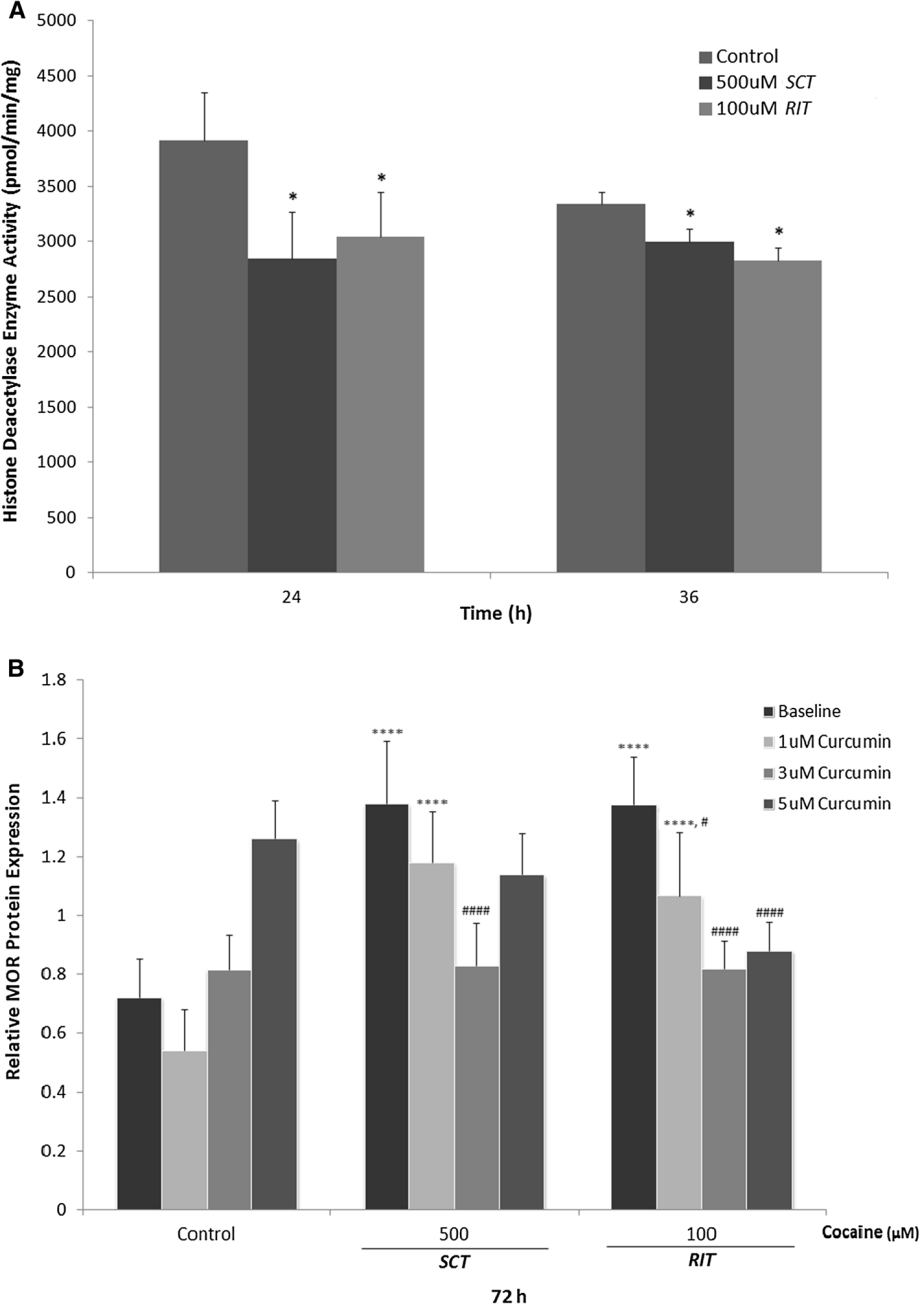
***Effect of cocaine on HDAC activity, and inhibition of HAT on cocaine-induced MOR protein levels.*** (**A**) Nuclear extracts were obtained from lysates of PC12 cells treated with cocaine (500 μM *SCT* or 100 μM *RIT*) for 24 or 36 hours. HDAC enzyme activity was determined using a fluorescent microplate reader and expressed as specific activity, in pM/min/mg. Statistical analysis revealed a significant decrease in HDAC activity following both cocaine treatment regimens at both 24 and 36 h. (**B**) Densitometric analysis of MOR protein levels relative to α-tubulin in control and 72 h cocaine (100 μM *SCT* and 500 μM *RIT*) treated cells grown in the presence and absence of 1, 3 or 5 μM curcumin. Compared to control, both *SCT* and *RIT* with cocaine increased MOR protein levels. For *SCT*, there was a significant decrease in MOR expression after pre-treatment with 3 μM curcumin compared to cells treated with cocaine alone. Pre-treatment with 1, 3 and 5 μM curcumin also prevented the increase in relative MOR protein levels resulting from 100 μM *RIT*. Results are representative of at least 5 independent experiments and data are presented as mean ± SEM (*p<0.05, ****p<0.0001 compared to control, #p<0.05, ####p<0.0001 compared to cocaine alone).

PC12 cells were treated with 500 μM *SCT* or 100 μM *RIT* cocaine, and HDACs activity in nuclear extracts was evaluated 24 or 36 h after treatment (Figure 4A). Following both time points and treatments, HDACs activity was significantly inhibited in comparison to control [F(2, 29)= 3.43, p<0.05]. There was no difference in the level of HDAC inhibition between either treatment conditions or treatment times.

In a subsequent experiment, PC12 cells were treated with 1, 3 or 5 μM curcumin, a non-selective HAT inhibitor, for 1h prior to exposure to either 500 μM *SCT* or 100 μM *RIT* cocaine for 72h. Relative levels of MOR protein from these cell extracts was compared to those obtained from untreated cells, and those treated with the inhibitor or cocaine alone. Statistical analysis revealed an overall significant interaction between the effects of curcumin and cocaine on MOR protein expression [F(6, 52)= 15.25, p<0.0001], a significant effect of curcumin alone [F(3, 52)= 20.62, p<0.0001], and a significant effect of cocaine alone [F(2, 52)= 15.87, p<0.0001]. Pre-treatment with 3 μM curcumin significantly decreased MOR expression in cells treated with 500 μM *SCT* cocaine compared to cocaine alone (p<0.0001). Additionally, MOR levels in cells pre-treated with 1, 3, and 5 μM curcumin followed by 100 μM *RIT* cocaine were significantly lower than those receiving *RIT* cocaine alone (p<0.05 for 1 μM, p<0.0001 for 3 and 5 μM curcumin).

## Discussion

The present study identified a number of cellular mechanisms by which cocaine alters MOR expression. Treatment of PC12 cells with either a single dose or repeated doses of cocaine increased MOR protein levels. The mechanisms regulating this increase were dependent on the treatment regimen used. Both *SCT* and *RIT* increased MOR protein stability, indicating that both regimens increased MOR protein levels post-transcriptionally. *RIT* elevated MOR and *c-fos* mRNA levels and AP-1 activity, but a single dose of cocaine did not, indicating that multiple cocaine doses were required for transcriptional regulation of MOR. Both dosing regimens also increased NO production and inhibited HDACs activity. Finally, cocaine-induced increases in MOR expression were attenuated by pretreatment with the NOS inhibitor L-NAME, and by the non-selective HAT inhibitor curcumin. Therefore, in PC12 cells, both NO and histone acetylation play an important role in the transcriptional and post-transcriptional regulation of MOR levels by cocaine.

Cocaine has been reported to increase MOR mRNA levels and receptor density in several regions of the rat brain [6,7,66]. In the PC12 cellular model, both repeated doses and a single dose of cocaine increased MOR protein levels, but only repeated doses elevated MOR mRNA levels. This suggests that different treatment regimens regulate MOR expression through different mechanisms, and that multiple doses of cocaine are necessary for transcriptional regulation of the MOR. To investigate this possibility further, the effect of *RIT* and *SCT* cocaine on potential transcriptional regulators of MOR expression, *c-fos* expression and AP-1 activity, was also assessed.

Several studies have demonstrated increases in c-Fos levels following cocaine administration [27,30,31,40,67]. c-Fos binds to members of the Jun immediate early gene family to form the AP-1 transcription factor [68,69]. Since the promoter of the MOR gene contains consensus sequences for binding AP-1 transcription factors [33], AP-1 activity was assessed in PC12 cells treated with cocaine. It was found that the effect of cocaine on AP-1 activity was dependent on the treatment regimen. In fact, only exposure to multiple cocaine doses increased *c-fos* mRNA levels and AP-1 activity, suggesting that increased MOP-r mRNA levels induced by 100 uM *RIT* in PC12 cells could be mediated by AP-1. Increased *c-fos* expression following repeated *in vivo* cocaine administration has been linked to cocaine-induced phosphorylation of CREB [70]. Since CREB is an important regulator of *c-fos* transcription (reviewed in [71]), it is possible the increased *c-fos* mRNA levels observed following RIT in the present study are the result of cocaine-induced CREB phosphorylation. Although we only focused on one potential candidate, other transcription factors, such as SP-1 and NF-κB are also increased in PC12 cells following cocaine administration. [72,73] and activation of these transcription factors has been linked to increases in MOR transcription and mRNA expression [74-76]).

The present study also investigated the role of NO in regulating cocaine-induced changes in MOR protein levels. NO has been found to contribute to the behavioral effects of cocaine, including conditioned place preference and sensitization [77-83]. In addition, NO has been reported to modulate CREB phosphorylation and DNA binding [84,85], increase the expression of immediate early genes such as c-Fos and Jun-B [25,28,29] and increase transcription from AP-1 responsive promoters [25,29], suggesting that NO could also be involved in regulating MOR transcription. In PC12 cells, pre-treatment with the NOS inhibitor L-NAME prevented cocaine-induced up-regulation of MOR mRNA and protein. Additionally, NO production was assessed visually by loading the cells with DAF-2DA as described previously [53]. Both 100 μM *RIT* and 500 μM *SCT* cocaine substantially enhanced DAF-2 fluorescence relative to control (untreated) cells indicating both dosing regimens result in increased NO production. This cocaine-mediated increase in DAF-2 fluorescence was blocked by pre-treated with 20 mM L-NAME, the dose of L-NAME that also prevented cocaine-induced increases in MOR protein and mRNA levels. Taken together, these findings indicate that NO modulates cocaine-induced changes in MOR protein levels. Interestingly, in *in vivo* neuronal models, cocaine increases NO production by increasing neuronal NOS (nNOS) protein levels and activation [22,23,78,79] via dopamine-, glutamate- and MOR-dependent mechanisms [22,23,79]. Because the PC12 cell culture model does not contain these pre- and post-synaptic systems, the current findings support the intriguing possibility that cocaine can alter NO levels and/or activity by direct intracellular actions. The specific target of these direct actions, and its biological significance, will require further investigation.

These experiments in PC12 cells identified another possible mechanism through which cocaine could increase MOR expression. NO has been reported to decrease HDACs activity [34,35], enzymes that are linked to the behavioral effects of cocaine in rats [36,37], and inhibition of these enzymes prolongs histone acetylation and contributes to enhanced transcription [43-47]. In the present study, both *RIT* and *SCT* cocaine decreased HDACs activity indicating that *in vitro* cocaine exposure enhances histone acetylation. In addition, pre-treatment with the non-selective HAT inhibitor curcumin prevented the cocaine-induced up-regulation of MOR protein levels for both dosing regimens. *In vivo* studies in rats have demonstrated that cocaine increases histone acetylation [36,86,87]. Our findings complement and extend these findings, and suggest that cocaine-mediated alterations in histone acetylation could be an important regulator of MOR protein levels. However, additional actions of curcumin could also contribute to these effects. In fact, cocaine induces the expression of cytokines, such as interleukin-1β [88] which can also regulate MOR transcription [89]. Curcumin inhibits interleukin-1β-induced NF-κB activation [90], suggesting the possibility that the anti-inflammatory properties of curcumin could contribute to its ability to inhibit MOR transcription.

The relationship between NO and HDACs is also complex. Although the cocaine-induced increase in NO observed in the present study could be responsible for decreasing HDACs activity, inhibition of HDACs has also been associated with increases in eNOS mRNA [91]. Increased constitutive NOS levels and increases in NO production resulting in S-nitrosylation have been reported to regulate protein stability [43,44,47]. Thus, in the PC12 cell model, NO and HDACs could modulate MOR expression independently, or may regulate each other to affect MOR transcription and protein stability. As well, the cocaine-induced increase in c-fos expression and AP-1 activity, only observed after RIT, together with increased histone acetylation could be involved in the transcriptional regulation of MOR. Interestingly, both cocaine and NO were reported to increase *c-fos* expression *in vivo*[92], and increases in *c-fos* are correlated with hyperacetylation of H3 histones during chronic cocaine administration and H4 histones after a single acute dose [93], suggesting that similar cellular mechanisms could be regulating the effects of cocaine *in vitro* and *in vivo*.

Although we identified NO and histone acetylation as important regulators of cocaine-induced MOR protein expression, how cocaine initiated the observed effects remains unclear. Blockade of sodium channels following passive entry of cocaine into PC12 cells is one possibility. However, this may not be the only mechanism because, in PC12 cells, NO activity is associated with extracellular signal-regulated kinase (ERK) pathway activation [94-97], and Tan et al. [98] found that the selective sodium channel inhibitor tetrodotoxin had no effect on this pathway. We determined previously that NO modulates ERK activity in PC12 cells [54], and since blockade of ERK phosphorylation in the nucleus accumbens of rats inhibits cocaine-induced behavioural sensitization [99], it is possible that in our system NO-mediated activation of ERK contributes to cocaine-induced MOR expression. *In vivo* cocaine exposure increases dopamine accumulation, through inhibition of dopamine reuptake by the dopamine transporter (for reviews, see: [14,100]), and the dopamine transporter inhibitor sydnocarb has been shown to increase NO generation [101]. Interestingly, Imam et al. [56] reported that cocaine, increased NFκB expression, and dose-dependently decreased both dopamine transporter expression and intracellular dopamine concentrations in differentiated PC12 cells. Elevations in extracellular dopamine could result in dopamine receptor activation, and inhibition of the D1 sub-type of dopamine receptor was reported to partially inhibit cocaine-induced increase in NF-κB [73]. Because NF-κB has also been implicated in regulating NOS expression and NO production [102,103], indirect activation of the D1 receptor by cocaine could enhance NO production. Whether the increases in NO observed in the current study resulted from cocaine-induced dopamine accumulation and dopamine receptor activation requires further investigation. Finally, cocaine has also been reported to diffuse through the membrane of cells [104], suggesting that it may exert direct effects on gene or protein expression.

There are several advantages to identify cellular mechanisms in cell lines, however some limitations of this system need to be considered. In contrast to neurons, PC12 cells continue to divide, and this could make them less vulnerable to neurotoxic compounds. Although we have identified many similarities between our findings and previous *in vivo* studies, differences in the biology of cocaine in the intact organisms compared to the effects observed *in vitro* are likely to exist. The physiological relevance of the doses and regimens employed in these *in vitro* studies is also not known. The doses selected for the present study were based on a previous study that characterized changes in proto-oncogene expression in differentiated PC12 cells [56]. It is not known whether similar treatment regimens, or doses of cocaine, would exert similar effects in an *in vitro* neuronal model or *in vivo*. Further the authors are not aware of any study that has established concentrations of cocaine in the extracellular fluid required to initiate changes in NO production or epigenetic modifications in neurons, or whether cocaine can act on these mechanisms by pathways that do not involve the transporters of catecholamines. Therefore, the current results in the PC12 cell model are important as they propose testable hypotheses about cellular and molecular mechanisms through which cocaine modulates MOR protein and gene expression in neurons.

## Conclusions

**Figure 5:**
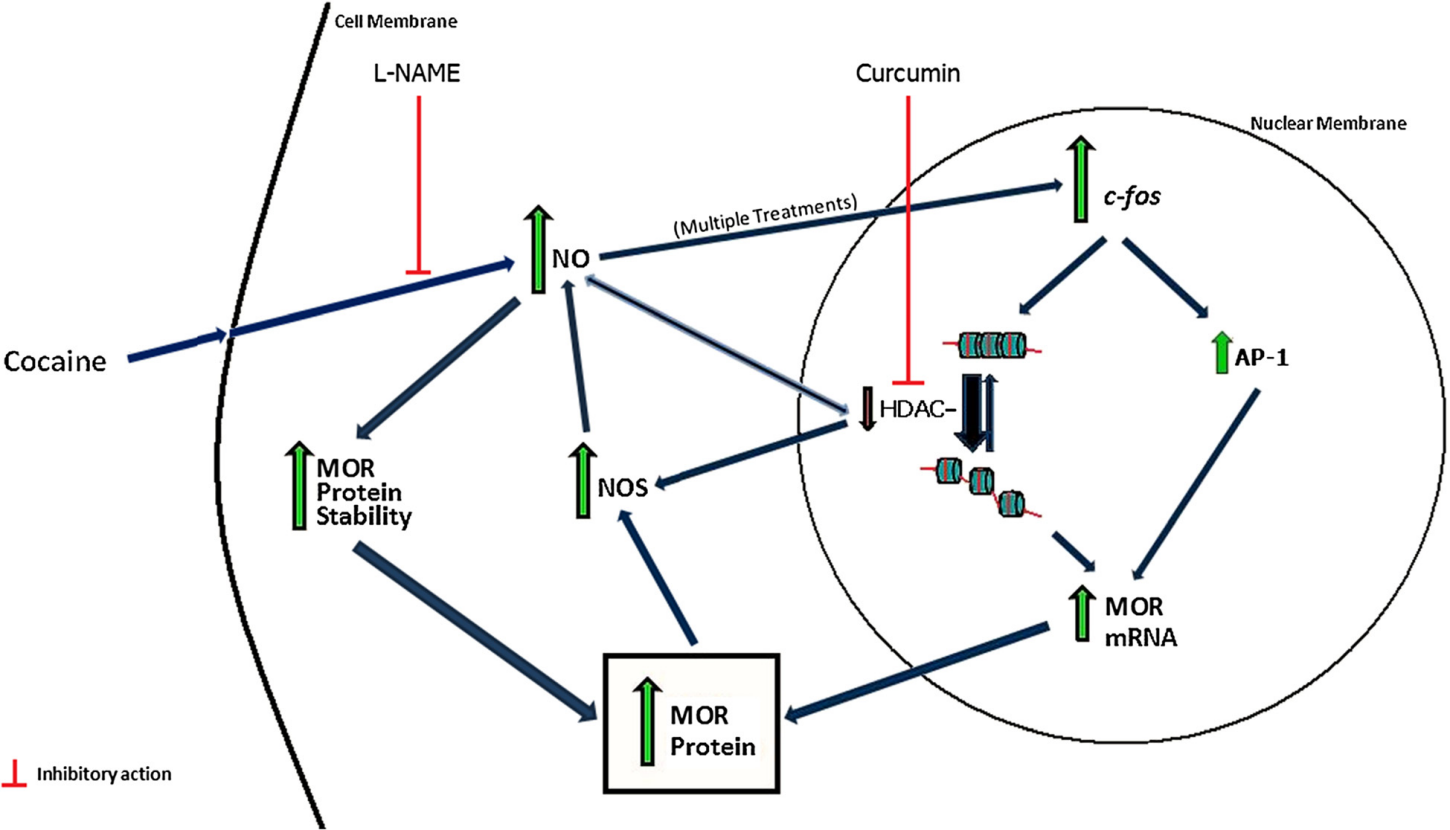
***Schematic representation of the proposed mechanisms for the in vitro action of cocaine on MOR expression.*** Cocaine increases NO production and decreases HDAC activity, which could lead to further increases in NO production as well as enhanced MOR protein stability. Multiple repeated treatments with cocaine also lead to increases in the levels of *c-fos* mRNA. c-Fos can bind to Jun transcription family members to stimulate AP-1 activity, which, in combination with HDAC inhibition, may contribute to an up-regulation in MOR mRNA levels through enhanced transcription. Sites of inhibition (⊥) by curcumin and L-NAME are indicated.

The data presented in this study support a model in which cocaine enhances MOR protein expression by increasing cellular NO levels and by decreasing HDAC activity (Figure 5). In addition to cocaine activating these effects independently, elevations in NO could also alter HDAC activity. Inhibition of HDACs could further enhance NO production, resulting in increased MOR expression and protein stability. Additionally, increases in *c-fos* (following multiple treatments) may lead to a modest increase in AP-1 activity that, in combination with enhanced histone acetylation, could contribute to the up-regulation of MOR transcription. Whether these mechanisms occur in neurons, and whether they have implications for the behavioral effects of cocaine, should be the focus of additional investigations.

## Abbreviations

ANOVA: analysis of variance; ANCOVA: analysis of co-variance; AP-1: activator protein 1; DAF-2 DA: 4,5-diaminofluorescein diacetate; HAT: histone acetyltransferase; HDAC: histone deacetylase; L-NAME: N^ω^-nitro-L-arginine methyl ester; MOR: μ-opioid receptor; nNOS: neuronal nitric oxide synthase; RIPA: radioimmunoprecipitation assay; SEM: standard error of the mean; TBS-T: TBS containing 0.1 % tween 20.

## Competing interests

The authors declare that they have no competing interests.

## Authors’ contributions

WW carried out all of the above experiments, performed the statistical analysis and contributed to the preparation of the manuscript. FL participated in the experimental design of the study, provided some of the materials used, and edited the manuscript. BK supervised the experiments, provided the laboratory facilities and the majority of the reagents used in the study, and contributed to the preparation of the manuscript. All authors participated equally in the conception of the study, the interpretation of the data and have read and approved the final manuscript.

## Pre-publication history

The pre-publication history for this paper can be accessed here:

http://www.biomedcentral.com/2050-6511/13/11/prepub
